# Qualitative Meta-Synthesis of User Experience of Computerised Therapy for Depression and Anxiety

**DOI:** 10.1371/journal.pone.0084323

**Published:** 2014-01-17

**Authors:** Sarah E. Knowles, Gill Toms, Caroline Sanders, Penny Bee, Karina Lovell, Stefan Rennick-Egglestone, David Coyle, Catriona M. Kennedy, Elizabeth Littlewood, David Kessler, Simon Gilbody, Peter Bower

**Affiliations:** 1 NIHR School for Primary Care Research, Centre for Primary Care, University of Manchester, Manchester, United Kingdom; 2 School of Nursing, Midwifery and Social Work, University of Manchester, Manchester, United Kingdom; 3 Mixed Reality Lab, School of Computer Science, University of Nottingham, Nottingham, United Kingdom; 4 Department of Computer Science, University of Bristol, Bristol, United Kingdom; 5 School of Computer Science, University of Birmingham, Birmingham, United Kingdom; 6 Mental Health & Addictions Research Group, University of York, York, United Kingdom; 7 School of Social and Community Medicine, University of Bristol, Bristol, United Kingdom; University of Stirling, United Kingdom

## Abstract

**Objective:**

Computerised therapies play an integral role in efforts to improve access to psychological treatment for patients with depression and anxiety. However, despite recognised problems with uptake, there has been a lack of investigation into the barriers and facilitators of engagement. We aimed to systematically review and synthesise findings from qualitative studies of computerised therapies, in order to identify factors impacting on engagement.

**Method:**

Systematic review and meta-synthesis of qualitative studies of user experiences of computer delivered therapy for depression and/or anxiety.

**Results:**

8 studies were included in the review. All except one were of desktop based cognitive behavioural treatments. Black and minority ethnic and older participants were underrepresented, and only one study addressed users with a co-morbid physical health problem. Through synthesis, we identified two key overarching concepts, regarding the need for treatments to be sensitive to the individual, and the dialectal nature of user experience, with different degrees of support and anonymity experienced as both positive and negative. We propose that these factors can be conceptually understood as the ‘non-specific’ or ‘common’ factors of computerised therapy, analogous to but distinct from the common factors of traditional face-to-face therapies.

**Conclusion:**

Experience of computerised therapy could be improved through personalisation and sensitisation of content to individual users, recognising the need for users to experience a sense of ‘self’ in the treatment which is currently absent. Exploiting the common factors of computerised therapy, through enhancing perceived connection and collaboration, could offer a way of reconciling tensions due to the dialectal nature of user experience. Future research should explore whether the findings are generalisable to other patient groups, to other delivery formats (such as mobile technology) and other treatment modalities beyond cognitive behaviour therapy. The proposed model could aid the development of enhancements to current packages to improve uptake and support engagement.

## Introduction

Common mental health problems such as depression and anxiety are highly prevalent and associated with significant personal and economic burden. Although these illnesses can be successfully treated with psychological therapies, there remains a significant disparity between need and provision[1]. This has led to a radical re-organisation of the traditional delivery of psychological therapies, with attempts to bridge the gap between supply and demand by encouraging patient use of health technologies with limited professional input in order to better manage limited therapeutic resources[2].Such health technologies are described as ‘low intensity’ treatment in the UK, typically referring to self care interventions with minimal professional input[3]. Computerised therapies are commonly employed as low intensity treatments, enabling patients to access therapeutic resources or support directly through remote technologies such as computers and phones. Barriers to accessing traditional face-to-face therapies such as time, stigma and cost mean that computerised therapies have a unique potential to contribute to extending mental health service capacity [4].

Computerised therapy typically refers to computerised cognitive behaviour therapy (cCBT) packages such as ‘Beating the Blues’ and ‘Fearfighter’, which are recommended in NICE guidelines for treating depression and phobic disorders respectively in England and Wales [5]. Computerised therapies can be delivered as entirely self-managed interventions or in conjunction with face to face or remote support. To date, the interest in computerised therapies has been driven predominantly by the need to increase access to psychological therapy, as it is less dependent on scarce therapist resources and can overcome logistical barriers such as time and travel. However, the acceptability of such interventions to patients has received less attention[6]. There are concerns that poor acceptability could limit uptake, and so limit the ‘reach’ of such treatments (the proportion of eligible patients that access them), and maximising the uptake of computerised health interventions is considered a priority area[7]. There has also been less attention given to the possibility that cCBT could raise unique barriers of its own. As well as concerns about the technical competence required of patients, novel technological interventions can be perceived to challenge perceptions of self and identity and disrupt existing routines [8]. Patients who are living with depression are recognised as going through ‘identity shifts’ as part of the trajectory of living with a chronic condition, which interact with how they view treatments and understand the meaning of interventions[9]. The impact of computerised delivery of therapy on such experiences is unknown.

The ‘first generation’ of mental health technologies have been developed to replicate existing methods of therapy, for example directly converting written self-help materials to online presentation, with less exploration of how technology could enhance therapy[10]. Beyond addressing resource and logistical demands, it has been suggested that computerised therapy could have positive consequences through empowering users to adopt a more active role in their recovery and to move authority and control in treatment from experts to service users or peers [11] [12].

There has been scepticism however about the potential for computerised therapy to be effective precisely because of the absence of therapist support[13]. ‘Non specific’ or ‘common’ factors (empathy, acceptance and the therapeutic alliance between therapist and client) are considered essential to effective psychological therapies, even more so than the specific content of a therapy model [14] [15]. Others have argued that the effectiveness of cCBT may be enhanced by the absence of such common factors, as these factors can aid therapy when positive but also interfere with therapy when less then optimal. cCBT therefore enables a standardised delivery of core treatment ingredients, for example behavioural activation or cognitive intervention, free from potential interference from other, more variable factors [16]. However, it is unclear whether patients themselves consider common factors essential, and the degree to which their absence is seen to impact on the credibility and effectiveness of cCBT, with little evidence around the role of user or carer involvement or experience in the development of computerised packages. The debate around user experience is also largely theoretical given the comparative absence of empirical research into patient experience of computerised therapies compared to studies investigating effectiveness.

The need to incorporate user perspectives into the design of interventions is increasingly recognised in health services research and is central to broad policy aims to deliver patient centred care sensitive to the views of patients[17]. Although there have been several qualitative studies exploring user perspectives, these have not yet been synthesised systematically, despite the critical role of such syntheses in providing a rigorous and comprehensive platform to guide evidence-based clinical practice. Existing reviews of cCBT acceptability and uptake have summarised qualitative reports [18] or examined attrition rates and quantitative survey data [19] but there has been no systematic review and synthesis of in-depth qualitative studies that can identify consistent or substantive themes in patient experience of cCBT to elucidate their impact on uptake.

The aims of the study were to:

1. Systematically identify relevant qualitative studies on user experiences of computerised therapy
2. Perform a meta-synthesis to identify common themes in user experience across studies and derive new insights from the synthesised data.
3. Discuss how such findings could contribute to the design of the next generation of computerised therapies.

## Methods

### Overview

The study had three stages: 1. Systematic search, 2. critical appraisal and 3. synthesis.

### Systematic Literature Search

The review investigated qualitative studies exploring user experience of using computerised therapies for anxiety and depression. The key search terms (and their truncated variants) were ‘mental health disorders’ (population), ‘technology assisted psychological therapy’ (intervention) and ‘qualitative research’ (outcomes/study design). Multiple search terms (Appendix S1), both MESH and textual, were derived from existing reviews of computerised therapy (the acceptability review by Waller & Gilbody[18] and the review of applications by Marks & Cavanagh[20]) and from a previous meta-synthesis of interventions for depression ([21]). Collaborators in the field of computer science and health services research (DC, SRE and CK) were also consulted specifically to ensure that terms appropriate to those fields were employed. Test searches were conducted and expert advice from specialists in retrieval was sought to maximise efficiency.

### Inclusion and Exclusion Criteria

Table 1 lists the inclusion and exclusion criteria. We focused on common mental health problems (anxiety and/or depression) managed in non-hospital settings, which are those most likely to be managed via computerised therapy. Other mental health conditions, including postnatal depression and posttraumatic stress disorder, were excluded as these disorders are typically deemed as more complex and requiring higher intensity or step 3 interventions. Technologies employed for these more intensive therapies (such as virtual reality for post-traumatic stress) were considered distinct from the technologies available to support primary care mental health and were excluded from the current review. We did not specify any model of therapy for inclusion (and therefore did not only include studies of CBT, although it was expected that these would form the majority of retrievals). We defined ‘psychological therapy’ using a modification of Strupp's definition as ‘a psychological process designed to bring about modifications of feelings, cognitions, attitudes and behaviour’[22]. Consequently, we excluded interventions which did not intend to promote therapeutic change (for example, websites used only for psychoeducation or for increasing awareness of mental health problems.) We also excluded papers in which participants' views were elicited about computerised therapy without direct experience of an intervention (one paper [23] was excluded on this basis.)

**Table 1:** Study Inclusion and Exclusion Criteria.

Inclusion	Exclusion
Peer reviewed journal articles or conference papers published between 2000–2012. Articles could be in any language and be published in any country	Unpublished dissertations, book chapters or papers published before 2000
Qualitative analysis reported. An operational definition of this criteria was that studies collected semi-structured interview data and undertake some form of thematic analysis	No qualitative analysis undertaken or primarily quantitative data reported. Questionnaire data and content analysis reports were included in this classification
Technology used to deliver psychological therapy	Therapy not delivered by technology. This included person-to-person therapy delivered by phone or video conferencing and technology used solely to support person-to-person therapy, e.g. using a mobile to record mood between therapy sessions
Therapy provided for anxiety or depression (with or without comorbid physical or health conditions)	
	Therapy provided for Post-Natal depression, Bipolar Affective Disorder, Substance Abuse (including nicotine), Dementia or other Cognitive Disorders, Eating Disorders, Psychotic Disorders or Personality Disorder.

The review focused exclusively on therapy *delivered predominantly or solely by* technology and excluded remote therapy *mediated by* technology (i.e. where the technology acts to facilitate direct patient-professional contact). Our interest was in the user experience of technologies as a low intensity intervention delivered with minimal or no professional support and in technology as a platform for delivering therapy in itself (rather than supplementing therapy delivered by a health professional, for example using a phone for patient consultations or using a computer only for information sharing between therapeutic sessions). Therefore, cases where the intervention was exclusively delivered by a health professional who used technology to communicate with patients were excluded. Cases in which limited contact with a professional was supplemented by independent interaction with a therapeutic technology were considered for inclusion.

We included papers which reported primary qualitative data collected through methodologies including interviews, focus groups, observation and ethnography (further detail on the inclusion criteria regarding this is included below in the ‘critical appraisal’ section.)

Five health science databases were searched during September 2012: Medline, CINAHL, PsychInfo, Embase and the Cochrane Library. Searches were limited to papers published after 2000 so that the technologies would be comparable (in terms of relating to current systems and widely available technologies) Additionally, key terms (‘Anxiety’ ‘Depression’ and ‘Mental Health’) were searched within the same date limits in a specialist computer science database, Association of Computer Machinery (ACM) Digital Library, to retrieve human-computer interaction literature which might not have been listed in health science databases. Additionally, in order to identify potentially eligible studies not published in traditional academic journals, the ACM special interest group Computer Human Interaction conference abstracts (2000–2012) were hand searched.

### Search Results

Duplicated papers were removed before screening. Study selection was conducted independently by GT. Ten percent of retrievals were reviewed by a second author (SK). Inter-rater agreement for full text screening was 98%. Authors were contacted for further information as necessary to clarify whether their paper met inclusion criteria (One author was contacted to determine whether user perspectives reported in their study were gathered through semi-structured interviews[24], The author reported that data was collected online as text commentary, and was therefore excluded).

**Figure 1:**
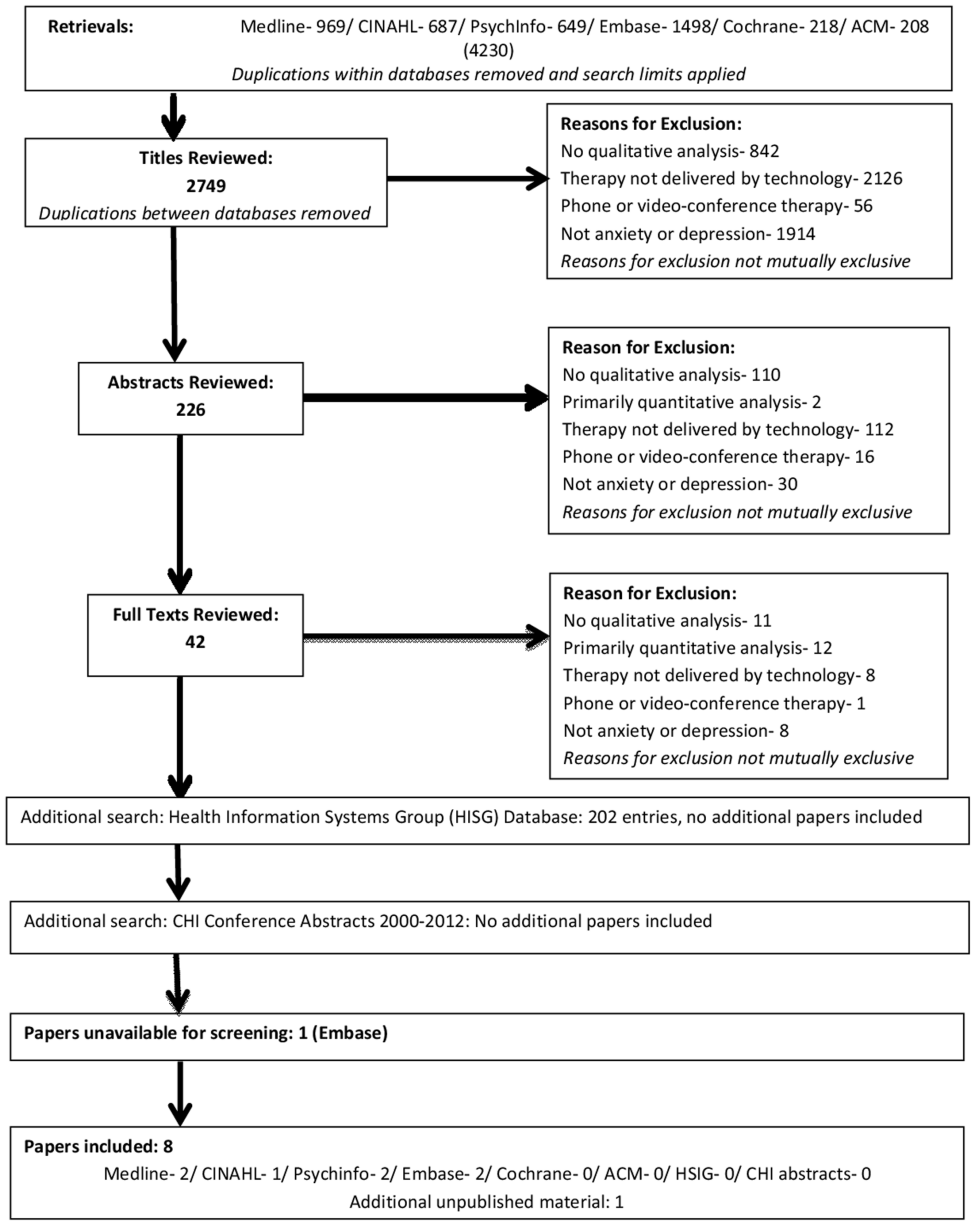
PRISMA flowchart.

Any disagreements about inclusion were resolved through recourse to a third author. Search outcomes are presented in the PRISMA diagram in Figure 1.

### Critical Appraisal

There remains a lack of formal consensus around the use of quality appraisals in qualitative reviews[25] [26]. For example, Atkins and colleagues reported that appraisal can reflect the written report rather than the study itself, but nevertheless found that the process helped to draw attention to which papers contribute most to the overall synthesis, with richer papers with ‘thick’ description and analysis tending to contribute more than purely descriptive papers[27]. To meet the aims of our synthesis, we were concerned more with richness of data than the rigour of studies, and therefore did not include or exclude papers based on quality appraisal, but did exclude papers that did not contain sufficiently ‘thick’ data. Thick data was defined as requiring at least semi-structured interviews for data collection and at least a thematic analysis of the data presented. Three papers were excluded as lacking sufficiently rich data, that is providing only descriptive data without a thematic analysis or collecting data through open response text rather than through interviews[28]–[30]. A fourth paper was excluded following consultation with the author as described previously[24]. Consistent with previous syntheses[31], we then placed greater emphasis on studies which included ‘thick’ descriptions, referring to in-depth examinations of user experience and provision of detailed and rich data, in contrast to ‘thin’ descriptions which lacked detailed description and did not further elaborate on reported experiences.

Two of the papers (Mitchell and Bradley) presented less primary data compared to the others, with only short illustrative primary quotations, and although they met inclusion criteria they were considered to contribute less to the synthesis itself. The Iloabachie paper, though meeting inclusion criteria due to collecting data through interviews and open comments, presented comments made during completion of the programme which appeared to reflect content (such as goal setting) rather than capturing user reflections about engaging with the programme. Ilobachie et al also discussed user experience within a conceptual framework (Theory of Planned Behaviour) but in relation to their mixed methods study, making it difficult to determine which second order insights emerged from the qualitative rather than quantitative data collected. Advocat et al provided a rich conceptual discussion but this was focused on a specific conceptual argument with minimal primary patient data provided. This paper contributed less broadly to the themes but did become relevant to the synthesis overall after reciprocal translation.

### Literature Synthesis

First order constructs were defined as direct participant quotes reported in the papers. Second order constructs were defined as the authors' interpretations of participants' quotes expressed as themes, extracted from both the results and discussion sections of papers in order to capture all constructs. Third order constructs refer to synthesised constructs that emerge from the analysis of first and second order constructs[27].

Papers were read and re-read by GT and SK and first and second order constructs were extracted and managed using Microsoft Excel. Extraction was checked by a third reviewer (CS). Constructs were reviewed to see how the themes juxtaposed and compared across papers. Reviewers independently sifted the second order constructs, compiling new third order constructs that summarised and encompassed the various themes across studies. Discussion with a third, independent reviewer (CS) then refined these constructs until a consensual understanding was reached.

Analysis followed the guidelines for meta-ethnography outlined by Noblit & Hare [32]. Noblit and Hare suggested three ways in which synthesis can be achieved; firstly through reciprocal translation, if the data is directly comparable, secondly through refutational translation, if the data is in opposition, and finally through a ‘line of argument’ which uses both similarities and differences across the studies to develop an integrating scheme, or a ‘whole’ that makes sense of the parts. Our reading of the included studies showed consistent themes but also apparent contradictions regarding users' experience of computerised therapy, and therefore the line of argument approach was utilised to make sense of apparent contradictions in the data and to integrate the emergent concepts to propose a model of user experience.

## Analysis and Results

### Search Results

Table 2 details the included papers[33]–[40]. Of the 8 studies included, 7 involved the use of desktop based computer platforms and 1 (Farzanfar et al) employed an automated phone system. The treatment in 6 of the studies was CBT. Of the remaining studies, Farzanfar employed adherence and self-care training and Iloabachie employed “behavioural activation, cognitive behavioural psychotherapy and interpersonal psychotherapy”. The participants in all studies were reported as experiencing depression, with the exception of the Mitchell and Bradley samples which included depression and/or anxiety, and the Advocat study which interviewed patients with panic disorder. The studies all included adult samples except Bradley and Iloabachie who interviewed adolescents. The other notable distinction was that the Hind study was the only study to specifically include patients with physical co-morbidities. Regarding study aims, the majority of the studies aimed to explore user experience of a mental health technology, with the exception of Advocat which aimed to explore user experience of being in trials of such technologies and focused more on the relationship between users and researchers, and how the use of mental health technology influenced users' perceptions of trials and treatment. Farzanfar also had a dual aim of exploring user experience to reflect on theories that users may be ‘anthropomorphising’ the system.

**Table 2:** Study characteristics of the eight synthesised papers.

	Reference & setting	Aims	Sample (age, ethnicity)	Patient group	Technology delivered therapy (& duration)	Additional support provided	Time point of data collection	Data collection method	Data analysis method
1	Hind et al (2012), United Kingdom	-Explore levels and determinants patient acceptability	4 men; 13 women, 30–61 yrs (median 46), ethnicity not stated	People with depression and Multiple Sclerosis	Beating the Blues or Mood Gym computer programmes (5–8 weeks)	No	After first session and at study exit	Interviews and written feedback	Framework analysis
2	Bradley et al (2012), Canada	-Find components that make intervention acceptable and effective, -Determine if Theory of Planned Behaviour guides adaptation	4 men; 9 women, 15–18 yrs (mean 16), ethnicity not stated	Adolescents, screened with the Depression And Anxiety Scale (DASS)	Feeling Better (Received 4 out of 12 modules)	No	Post intervention (At end of 4 modules)	Semi-structured interviews	Inductive thematic analysis
3	Gerhards et al (2011) Netherlands	-Explore patients experience and reasons for attrition	9 men; 9 women, (mean 43.6 yrs), ethnicity not stated	People with depression	Colour your Life computer programme (8 weeks)	No	Up to one yr from start of study	Semi-structured interviews	Grounded theory
4	Ilobachie et al (2011) USA	-Describe adolescent and parent experiences, -Establish knowledge base	Overall sample 36 men; 47 women, 14–21 yrs (mean 17.4), 38% Caucasian, 23% African-American, 5% Hispanic, 6% Asian, 4% other	Adolescents with depression	Cognitive Behavioural Humanistic and Interpersonal Training computer programme, (6 weeks)	Yes- one face-to-face contact and up to two telephone calls	Pre, during and post intervention	Interviews and typed comments	Grounded theory
5	Advocat & Lindsay (2010) Australia	-Explore experience of participant in internet-based mental health trials	2 men; 8 women, 20–66 yrs (mean 45), 9 Anglo-Australia, 1 Chinese-Australian	People with panic disorder	Cognitive Behavioural Therapy for Panic Disorder computer programme (12 weeks)	Yes- email support	At trial completion	Semi-structured interviews	Two step coding method
6	Farzanfar et al (2007) USA	-Explore attitude to IVR system, -Evaluate the development of anthropomorphic relationships	6 men; 9 women, 20–60 yrs, 2 Hispanic, 5 Black, 8 White	People with depression attending psychiatric clinic	Automated Telephone Linked Communication for Depression, (4 weeks)	Yes-psychiatric clinic appointments	During final three weeks of intervention	In depth interviews	Thematic analysis
7	Mitchell et al (2005) Australia	-Elucidate model of therapy and the role of computers in model	1 man; 7 women, 19–62 yrs (mean 39), ethnicity not stated	Primary care patients with current/past depression and/or anxiety	Climate Panic Online computer programme, (4–5 weeks)	Yes- Group Therapy	Post intervention	Semi-structured interviews and participant observation	Grounded theory
8	Knowles et al (Unpub*), United Kingdom	-Explore experiences of cCBT	11 men; 25 women, 29–69 (mean age 51), All White British except 1 White Other	Primary care patients with depression	Mood Gym or Beating the Blues computer programmes (6–8 weeks)	Technical support	Post intervention	Semi-structured interviews	Thematic analysis

* Data available on request

The synthesis revealed two core constructs, which taken together enabled us to derive new insights regarding barriers to, and potential facilitators of, engagement with computerised therapies. These were:

1. The desire for computerised therapies to be responsive to ‘self’
2. The dialectal nature of user experience

### 1. Programme sensitivity to ‘self’ and identity:

A consistent theme regarding the desire for programmes to have greater sensitivity was apparent across 7 of the 8 studies. Analysis across first and second order constructs demonstrated two components to this (presented in table 3); firstly, sensitivity to ‘Who I am’ as a patient, including different clinical needs (such as physical comorbidity) and personal preferences (with users requesting reactive, personalised content) and secondly, sensitivity to ‘How I Feel’, recognising the demands of depression on the user (such as emotional and motivational difficulties, and problems with concentration). In terms of implications of computerised therapy for understanding of ‘self’, this finding suggests that currently it is the *absence* of self which is most prominent in user experience of computerised therapy.

**Table 3:** Examples of 1^st^ and 2^nd^ order constructs and synthesised themes.

Study	1^st^ Order	2^nd^ Order	Synthesised theme
***Farzanfar et al***	“I like it when he says my name. I didn't like it when he didn't say my name”	Users were pleasantly surprised when the system remembered and referred to previous conservations and this facilitated greater connection	Need for computer to be sensitive to ‘Who I am’:Personalised material, responsive to the individualRelevant material, rather than generic examplesAppropriate to specific clinical needs, for example co-morbidity
***Gerhards et al***	“There were often things that I never had any problem with, then I thought this has nothing to do with me”	Self-identification…was a motivator towards adherence… many had difficulties translating and applying homework assignments to their own social situation	
***Knowles et al***	“when they were relevant to me it was fine, you know, but when they weren't it was so frustrating”		
***Hind et al***	“If you are really targeting it specifically at people with MS maybe it would be helpful to look at how people manage when they've got [disability and fatigue]. You know… being realistic about what you can do”	CCBT packages did not acknowledge the interaction between physical illness and their depression.	
***Farzanfar et al***	“It was really kind of…-do not be so cheery about the fact I am about to jump out of a 30th floor window!”	Participants felt response was not sufficiently ‘sensitive’ or ‘human’. It needed to convey … a sensitive tone that indicated compassion and concern	Need for computer to be sensitive to ‘How I feel’:Sympathetic or empathetic content, awareness of the difficulties faced.Appropriate for someone experiencing the low mood and low motivation typical of depression
***Knowles et al***	“to come and have to do something on a computer at night which I deemed as work, my mind automatically saw as work and effort…and the amount of motivation that it takes when you're depressed to go and do work it just doesn't seem to add up at all”	Participants referred to the demands of completing treatment and how this was a struggle particularly for depressed patients	
***Bradley et al***	“it's just black and white…it's like you're doing homework…' 'people will just kind of get like ‘ugh’ because they're already feeling like not very happy and then it's all grey and stuff”		

The sensitivity theme was absent in the Advocat paper. This may be because that study aimed to explore experience of being in cCBT trials (rather than direct experience of the intervention itself). The theme was most explicitly discussed in the Farzanfar paper, which may be due to the authors' aim to examine whether participants anthropomorphised the system, and so focused on attributions of reactivity and sensitivity in comparison to an actual human interface.

The expectation that programmes should respond sensitively may be due to users experiencing computers as social agents. Farzanfar in particular elaborated on this issue, describing how participants spoke about the system as a professional actor, for example using personal identifiers such as “he” and reporting they continued to engage because they “didn't want to disappoint” the computer. This is supported by data from two of the other studies; Hind et al also referred to the interpersonal reactions that users had to the programme, becoming “angered and frustrated” when the system was “insensitive” to their situation, and Knowles et al also reported participants responding to the computer as an actor: “I felt that the computer cared, and I know that sounds absolutely ridiculous, but it was like speaking to somebody but different”.

The experience of computers as agents is a significant topic of interest in Human Computer Interaction (HCI) literature. Reeves and Nass, together with collaborators, have undertaken an extensive body of research on the ‘Media Equation’[41]. This work suggests that people can treat computers as social *actors* and respond to computers and other media in ways derived from their response to other people. The creation of user interfaces which include embodied agents[42], [43] - photographs or animated characters - is one example of an explicit attempt to co-opt this response as a design resource that engages users and improves the interaction experience. The question of how to effectively design such interfaces for a given context, and the degree to which people do treat computers as social actors, is a subject of ongoing research and debate within the HCI community. In recent years researchers such as Bickmore and Gruber have provided initial evidence of the potential of such interfaces in mental health contexts[44]. Such work could provide valuable insights for the development of future CCBT services, to design interfaces which better capture the interaction between user and computer and exploit this to provide sensitised feedback to users.

This first construct of sensitivity relates directly to the second, which concerns the dialectal nature of user experience, with greater sensitivity one potential way of reconciling the conflicting perceptions. This will be discussed further in the following section.

### 2. Dialectal nature of user experience

Further analysis of the constructs revealed a pattern of dichotomies or contradictions were present in user experience regarding the level of support and contact with others when using technologically delivered therapy (further examples are given in Table 4).

**Table 4:** Contrasting positive and negative user experiences (Data in italics indicate first order data).

Positive User Experience	Negative User Experience
***Empowerment***	***Burden***
Gerhards: *The ability to solve it yourself, I think that*'*s a big advantage of such a course. That you can do it in your own way.*	Gerhards: *I just thought: I*'*m just torturing myself, I've had enough, I don't want this anymore (*…*). To write this feeling down and then at the end of the day to think about how I felt. It made me even more depressed*
Iloabachie: [participants] appreciated the control they experienced during the program/many adolescents…appeared to shift from passive to pro-active problem solving	Iloabachie: [participants] found the reading and skill builder assignments lengthy and tedious to complete, despite extensive revisions to reduce such burdens
Advocat: Some participants found the discipline required of the online trial freeing	Advocat: The freedom of choosing the right expert and engaging in treatment from her own home, in her own time, was sometimes difficult. Anne wanted not to be understood as simply a consumer, but as a client, a patient even, a person needing help from an expert.
Knowles: *Rather than just saying well here's your pills or sit there and talk to somebody for 35 minutes*…*actually felt like I was doing something to help myself*	Knowles: *To come and have to do something on a computer at night which, my mind automatically saw as work and effort*… *the amount of motivation that it takes when you*'*re depressed to go and do work*
Mitchell: *I didn*'*t know what to expect from the group but the computers in the room gave me a bit more confidence because I thought ‘I can do that/I felt good because I could do it*	Hind: *You still had to try and come up with the problems yourself and that*'*s quite difficult and I found it quite stressful* … *I mean, it*'*s hard doing it yourself*
***Anonymity***	***Burden***
Gerhards: *Here I drop my mask. And I only had that when I sat alone behind the computer, this is me, this is how I feel, and I experience it like this*	Gerhards: *I need to be with people, I can*'*t just be alone behind my computer screen*
Knowles: *Maybe because I wasn*'*t speaking to somebody, it didn*'*t hurt me to write down my feelings*	Knowles*: You do feel very alone*… *[working on the computer] sort of highlights it*
Mitchell: *I felt comfortable one-on-one with the computer, headphones on and in your own space*.	Hind: Y*ou can become very isolated because of your disability* … *So, I think when working with something that is a computer programme it makes you feel even more like you*'*re not speaking to someone face to face*.
Bradley: Y*ou can kind of do it in a secluded area where nobody is watching you*…*the privacy is kind of like a really big appeal*	

These contrasts therefore related to level of support and also to level of contact with others. Regarding support, two papers refer to the ‘empowering’ nature of computerised therapy (Ilobachie et al, Knowles et al) and a third to the sense of ‘mastery’ conferred on users (Mitchell et al). Gerhards et al reported that completers in their study tended to tailor the programmes to their own needs by selecting only those components perceived as beneficial, again reflecting a sense of ownership or control. The disadvantage of this independence was also apparent though, with computerised therapy perceived as burdensome (Hind et al, Knowles et al) and challenging (Iloabachie et al), placing great demands on the user, linked to both the additional responsibility of needing to tailor the generic materials to oneself and also to the absence of support from others. Advocat et al give an example of a participant who found the responsibility “confronting”, as the flexibility of the programme demanded a level of self-discipline to ensure completion. The following quotes illustrate clearly this distinction between perceptions of computerised therapies as ‘enforcing autonomy’ whereby work is typically undertaken without help and perceptions of therapy as ‘providing control’ whereby the active nature of cCBT is embraced.

“You…had to try and come up with the problems yourself and that's quite difficult and I found it quite stressful … I mean, *it's hard doing it yourself*” Hind et al, italics added.

“Rather than just saying well here's your pills or sit there and talk to somebody for 35 minutes…actually felt like *I was doing something to help myself*” Knowles et al, italics added.

A similar contrast is apparent regarding level of contact with others. Hind et al, Gerhards et al and Knowles et al all reported that participants expressed a need for human interaction to support use of the programme, and the sample in Ilobachie et al felt the programme would benefit from increased interaction between peers. However, the benefits of the absence of face to face or personal contact or the benefits of greater distance are also emphasised by users – Gerhards et al, Bradley et al, Mitchell et al and Knowles et al all refer to the advantages of engaging in therapy without direct or face to face contact, as it could confer a sense of personal safety and freedom to disclose. Again, the dichotomy is powerfully illustrated in the following quotes – from the same study, with users of the same programme, which emphasise the freedom and self-affirmation of anonymity on the one hand compared to the another participant for whom this means they are “just alone”:

“Here I drop my mask. And I only had that when I sat alone behind the computer, this is me, this is how I feel, and I experience it like this” Gerhards et al, italics added.

“I need to be with people, *I can't just be alone* behind my computer screen” Gerhards et al, italics added.

We analysed second order constructs to explore whether these competing attributes were explicitly conceptualised in any of the included studies. The contradictions do appear to have been recognised by the study authors, although they are not the main focus of the papers. For example, Hind et al referred to the “the trade-off between privacy and social isolation”. Iloabachie et al comment that internet therapies would need to “balance several strengths and limitations”. Gerhards et al commented that participants expressed a desire for support, but that this may counteract other elements of cCBT that were appreciated, stating that “adding support might endanger the appealing aspects of CCBT”. Knowles et al presented primary data on a continuum, showing that users could have either positive or negative experiences within the reported themes.

The most explicit discussion of this contradictory nature of user experience was within the Advocat paper, which aimed to examine whether participants in online trials could be viewed as ‘autonomous consumers’. Although their data considered participants' relationship to researchers, their analysis does have similarities with the contrast being patients as ‘passive’ or ‘empowered’. They reported that “Some participants…had more difficulty finding the balance between depending on the researchers and actively taking responsibility for their recovery.” They identified computerised interventions as being particularly responsible for this; “We identify the continuance of an important shift with the use of the Internet to conduct RCTs in which the individual is called upon to be more autonomous”, but reported that participants still felt a conflicting need to be dependent and treated as a patient.

Two of the papers conceptualised these contradictions as reflecting that different patients would experience computerised therapies as positive or negative. This formulation, which views the responses as reflecting different patients rather than different aspects of the same patient experience, would have significant implications for targeting such interventions. Mitchell et al for example commented that “people respond differently to varying types of interventions” and “computer-assisted therapy is not universally appealing or beneficial for all group members.” Knowles et al also referred to targeting computerised interventions, suggesting that users with the most negative experiences engaging in ‘deliberate non adherence’ as the intervention was not acceptable to them, whereas more positive users could experience benefits, which again conceptualises these contradictory reports as due to different patients having contrasting experiences.

Contrary to the different responses reflecting *different* patients' experiences, it may be that the *same* patients could experience both aspects of the programmes depending on the specific interface of the programme or potentially varying due to clinical factors, such as mood. However, given the available data, it is not possible to conclude whether the contradictions represent distinct groups of patients or whether the same patients reported both elements, and this should be explored further in future research.

**Figure 2:**
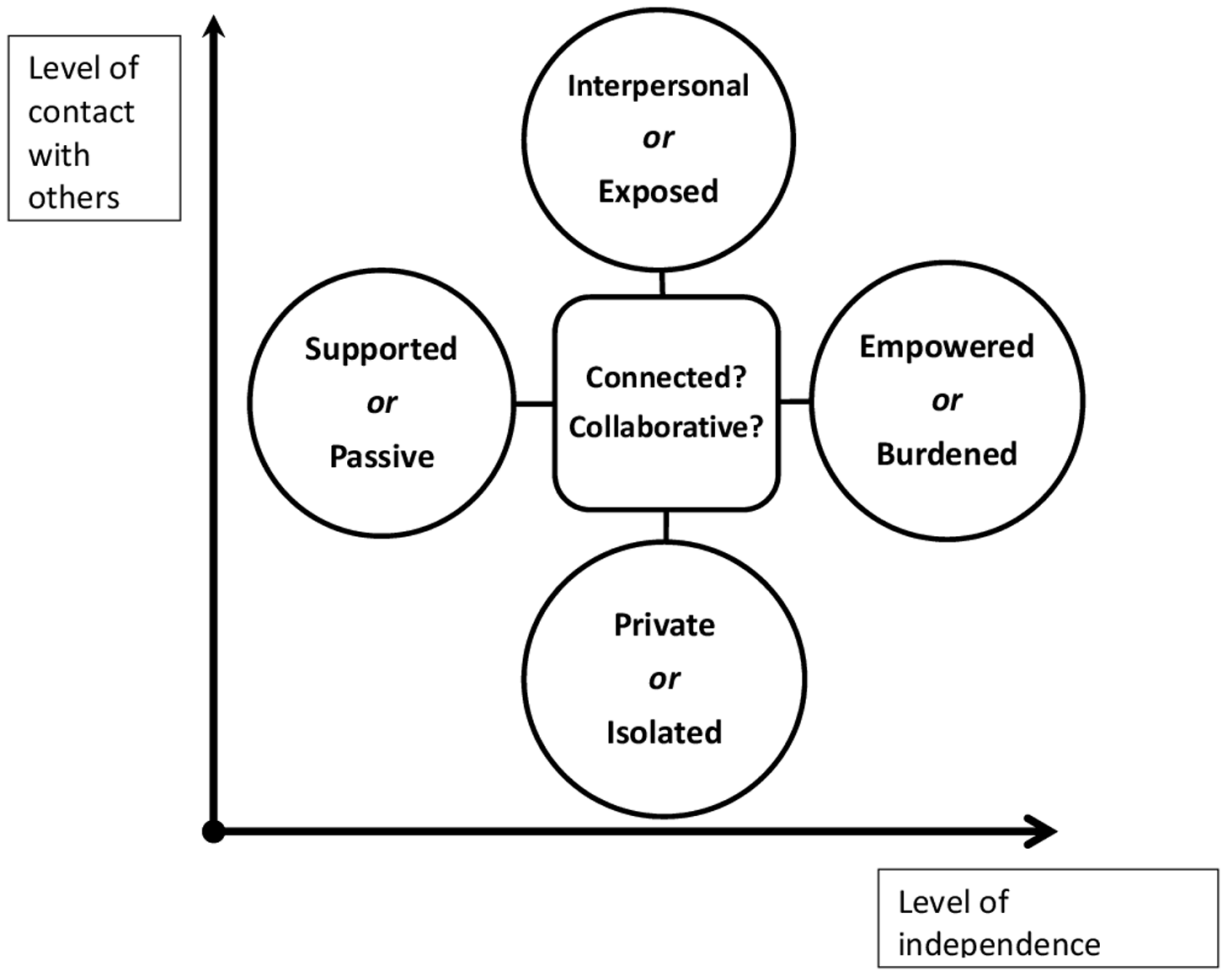
Dialectal representation of the experience of computerised therapy and potential unifying constructs.

Nevertheless, it was clear that the ‘same’ aspects of computerised therapy could be portrayed as both positive and negative experiences, rather than there being exclusive barriers or facilitators. The dialectal nature of experience was therefore most clear when themes were synthesised, using the line of argument approach, across the studies. Although secondary data showed that study authors were also aware of this tension, the line of argument analysis allowed us to explicitly conceptualise these contradictions to combine findings across the studies, integrate this with the identified construct of ‘sensitivity to self’ and develop new insights (Figure 2). Specifically, if it is the case that the same users experience both the positive and negative extremes expressed in Figure 2, then designing a system that balances these extremes may provide ‘the best of both’ and best support engagement. If it is the case that the extremes reflect the contrasting experiences of different patient groups, then it is possible that more negative patients could be supported to have a better user experience through designing systems which achieve a middle ground between the two extremes.

Figure 1 presents the dimensional themes relating to content and support, showing that both high and low levels of each of these dimensions can be experienced as either positive or negative. The greater level of contact confers benefits of interpersonal interaction, but can also be threatening. Conversely, a lower level of contact provides safety and privacy, but can also feel isolating and lonely. Regarding the level of independence, a high level of autonomy is perceived as empowering, but can also be experienced as too demanding. A lower level of independence means that more support is experienced, but in current systems limits the role of the user as an active agent, making them more passive, rather than having ownership over the process.

This conceptualisation allowed us to define the characteristics of a system that could balance these competing needs. Firstly, this computerised therapy programme would foster an experience of **collaboration** between the user and the system (which may include a sense of collaborating with peers or experts involved in the intervention). This would be consistent with the idea of ‘autonomy support’ [45]which emphasises the need to foster individual action to encourage self-motivation, rather than either providing solely external motivations or pressures, but also distinct from a feeling of ‘enforced autonomy' as present in the data sets, where users felt abandoned to cope alone or overburdened by the absence of support.

Secondly, the programme would encourage **connection**, balancing the need for contact with others (through either actual interaction or felt identification) without intruding on the private experience of the user. This could involve capitalising on the unique potential for computerised therapies to allow greater distance and even anonymity but also greater interaction through connection with online networks, or through supporting synchronous or nonsynchronous communication with therapists through ubiquitous technology such as text, video messaging or email. I

Both these concepts are integrated with the need to recognise self and identity; both aspects of sensitivity relate to enabling users to better connect with the programmes (through providing relevant content or identifiable examples) and to achieving a more collaborative relationship through recognising the needs of that particular individual. It is notable that the Hind paper was the only study to report only the negative attributes of computerised therapy and not report user empowerment or appreciation of privacy. The authors focused on the inappropriateness of some of the programme's material for patients with multiple sclerosis. The failure to identify with the programme may have resulted in a predominantly negative experience with patients experiencing only burden and isolation. The other included studies also provided examples of participants or study authors indicating that greater interactivity could increase the sense of collaboration and connection to the programmes. Iloabachie et al referred to “personalization and interaction… offering the promise of greater engagement”. Gerhards et al commented that “experiencing self-identification … was a motivator towards adherence.” A patient in Knowles et al commented: “when it wasn't relevant…that almost burst the bubble and broke the spell. I would step away from it and think no, that's not me”. Increased sensitivity to the user may therefore be necessary to maintaining a sense of connection and fostering a feeling of collaboration.

## Discussion

Providing CBT through computers has been driven by resource demands and a need to improve patient access to therapy rather than a focus on the patient experience. However, there has been increasing recognition that computerised formats could offer novel therapeutic experiences, beyond being merely the most accessible delivery mode. The meta-synthesis presented here revealed the distinct benefits that computerised therapies could have compared to typical face to face delivery, including more privacy and a greater sense of mastery and control, but also demonstrates that each of these benefits can also be perceived as a limitation in contrast to face to face therapies. We propose that balancing the perceived competing needs of support or independence and privacy or interconnectedness could enable more users to exploit the unique benefits of computerised therapy whilst maintaining gains in access and cost effectiveness. The meta-ethnography also made clear the importance of systems being sensitive to users, tailoring and personalising content to be most relevant to them, and indicated that this in itself could enhance feelings of connection and collaboration.

Our findings are consistent with sociological accounts of technology which argue that “technology-in-practice” approaches are necessary to understand the interaction of health and new technologies[46]. Novel technologies are viewed as mediators in constructing new social, personal or professional roles and identities, requiring in-depth qualitative research to explore the consequences of such change. In the case of mental health, computerised self-help therapies such as cCBT may be changing the role of patients in their own treatment, which can have both positive and negative consequences for patients. Negotiating new ways of providing peer or professional support and better understanding the role of patient identities in interacting with computerised therapies may be critical to achieving their full potential. Similarly, the impact of depression and its treatments on perceptions of self is a recurring theme in social research on illness experience (9). Users in the studies included in the review appeared to struggle with the absence of reflected or identified self in current computerised therapies, and incorporating greater sensitivity to patient identity may be crucial to support engagement.

### Limitations

Although we deliberately excluded interventions in which therapy was mediated by technology in order to examine experiences specific to receiving a therapy from technology, it may be that such mediated therapies share issues common to all technologies or that differences between the two helps elucidate further relevant themes. Particularly given the emphasis in the synthesised findings on including human support, future research should consider whether there are specific issues relevant to integration of health professional input with computer delivered content.

One study in the synthesis involved an automated telephone programme, but all others dealt with desktop based computerised platforms. Although this is reflective of those programmes currently in use in the NHS (Beating the Blues, FearFighter and Living Life to the Full), this nevertheless shows that little is known regarding the acceptability of other formats, such as mobile phone applications or text-messaging interventions. Such platforms may pose novel barriers of their own and the findings of the synthesis may not generalise across technologies. The specific criteria chosen reflect the difficulty of defining ‘computerised therapy’, which can include multiple types of technologies, different levels of professional or peer involvement and varying treatment aims, ranging from increased awareness to psychoeducation and delivery of evidence based therapies themselves. We hope that this illustrates the need for future research to consider the heterogeneous nature of mental health technology and explore whether barriers and benefits are consistent across the different types and formats. Attempts to create ontologies of such systems, for example those proposed by Coyle and Doherty[47], may be useful here.

The review was not performed using double screening, with one author (GT) performing the review and only subset of the studies reviewed by a second author which may be considered to methodologically weaken the study. However, the decision to do this was based on the high inter-rater reliability observed in a randomly selected double screened subsample which reassured us that the screening methods and criteria were adequate.

Our final analysis suggested that connection and collaboration could offer a way of reconciling the apparent contradictions of user experience. Platforms which may be most typically associated with increasing connection and collaboration, for example peer to peer networks or support forums, were excluded from the review if there was no explicit delivery of therapeutic content. Future reviews should explore the evidence base for mental health user experience around the use of social media and interactive technologies. However, the finding that users experience the computer or other device as actors suggests that connection and collaboration could still be fostered through typical desktop platforms, by exploiting insights into ‘computers as agents’ from the HCI literature.

We did not find significant differences in experience between adult and adolescent samples; however, as the two papers with adolescent samples were considered to contribute less to this synthesis, this may reflect a lack of in depth data exploring adolescent experience available for the review. The review also demonstrated a lack of qualitative research into the experience of diverse patient groups, particularly older people, black and minority ethnic groups and populations with mental and physical co-morbidities. The findings presented here may not translate to these groups, and exploring user experience in these populations should therefore be a key target for future research. The review was also limited primarily to CBT therapies. Other types of therapy may lead to different reactions from users when presented online, and it cannot be concluded on the basis of the current evidence that the user experience factors are independent of the therapeutic model employed.

### Implications

Whether cCBT is enhanced or impeded by the absence of the ‘non specific’ or common factors present in face to face therapeutic encounters has been debated in the literature. Our analysis suggests that this debate neglects a third possibility, wherein computerised therapies have additional and unique common factors themselves, relating to the particular properties of therapy delivered by technology such as privacy and control. The delivery of future technologies may not be best served by attempts to mimic the attributes of typical therapy, but might aim to exploit the common factors of therapy delivered by technology.

It is possible to draw a distinction between ‘complementary’ and ‘emulating’ approaches to technologies. Emulating approaches attempt to use technology to replicate something that exists between human actors, for example mimicking a human therapist. Complementary approaches, by contrast, consider what the technology offers that is different. The conceptualisation of common factors of computerised therapy would be consistent with a complementary approach, focusing on what technology can provide that is novel to the medium rather than trying to replicate what human therapists would do. This perspective would also support the framing of computerised therapy as complementary to face to face support, rather than replacing it. Computerised therapy can be delivered with varying levels of support, and there is some evidence that support is associated with better outcomes[20]. Greater connection and collaboration could be achieved through achieving a balance of independent computerised therapy with some level of professional support. However, attempts to increase provision of traditional support would need to maintain the cost-effectiveness of reduced therapist time. In technological interventions for chronic illness, Rossler and colleagues noted that the most ambitious therapies tended to be those with complex combinations of automated technologies and therapist involvement and the development of such combinations was key for future research[48]. This challenge is likely to also apply to the mental health technology field. An alternative approach to increased professional involvement would be to explore the use of peer support, but the acceptability of online social interventions for mental health is unknown, although third sector organisations are increasingly employing such platforms to provide mental health support (for example, the Elefriends network hosted by the UK charity MIND).

The finding that users experience the computer as a social agent does however suggest that collaboration and connection may be possible through improved interfaces – that patients may be able to achieve a collaborative relationship and connect with the computer therapy itself. In particular, greater personalisation of material to ‘Who I am’ and ‘How I feel’ could encourage engagement and interaction. Computerised formats are vastly amenable to personalisation [49] [50]but the present review indicates this potential remains untapped. Further research is necessary to determine whether improving user experience through these methods is possible. However, it is important to note that the synthesis cannot exclude the alternative suggested by two of the included papers, namely that different users will consistently experience computerised therapy as positive or negative. This should also be further explored, examining whether users can be reliably identified as positive or negative or whether experience can vary within users (for example, dependent on mood or severity of depression). If supported, this would have different implications for service delivery, indicating that computerised therapies need to be appropriately targeted and matched to specific users depending on patient preferences or aptitudes.

The suggestion that connection and collaboration can improve user experience has similarities to the design strategies proposed by Doherty and colleagues [47] which recommended that online mental health interventions be ‘interactive’, ‘personal’, ‘supportive’ and ‘social’. Greater collaboration with experts in computer science and human computer interaction is likely to be necessary to fully exploit the potential of computerised therapies, and in particular drawing on expertise in interactive design and user engagement may be especially valuable. One of the papers included in the present study, by Advocat and Lindsay, warned that the greater autonomy and control given to patients in online therapies means they have “the power to turn off the computer.” Although all therapies require engagement by the patient to be effective, computerised therapies require a more active involvement and motivation to engage in the therapy at all. Future research should draw on the design sciences and on the expanding field of human-computer interaction to better understand how to motivate users to engage with online treatments. Recent innovations such as Experience Based Co-design emphasise the importance of including users to specifically enhance quality of care and improve outcomes[51]. Such principles are also integral to the design sciences, including the field of human-computer interaction, and so such methodologies may be particularly important for guiding future development of technologies in health research. The methods of data collection included in the final synthesis were almost exclusively limited to semi-structured interviews, and it is likely that more in-depth, observational data collection methods will be necessary to better capture user experience in future. Ethnographic methods are considered particularly useful in the field of HCI, as they allow designers to capture how technologies are used ‘in the wild’, and so better understand the needs of users.

## Conclusions

The meta-synthesis enabled us to synthesise across the available literature to produce overarching recommendations for future service design, whilst maintaining the richness associated with individual qualitative studies. Mapping the identified themes across the studies allowed us to observe the dialectical nature of the perceived benefits and limitations of computers for providing mental health treatment. We modelled these contrasting attributes to identify the potential middle ground that could reconcile apparently contradictory positives and negatives regarding experience of computerised therapy. Future research should explore the potential of modern technologies to foster a sense of collaboration and connection, with peers, professionals or computer agents, in order to improve engagement with computerised therapy. Expanding the evidence base to consider diverse patient groups and working with experts in the design sciences will be necessary to fully achieve the potential of computerised therapy as a mental health treatment.

## Supporting Information

Checklist S1 **PRISMA Checklist.**
(DOC)

Appendix S1 **Search Terms.**
(DOCX)
