# Supplementary material for: Qualitative Meta-Synthesis of User Experience of Computerised Therapy for Depression and Anxiety
Source: PLoS One. 2014 Jan 17;9(1):e84323. doi: 10.1371/journal.pone.0084323 (PMC3894944; doi:10.1371/journal.pone.0084323)
Supplement: Appendix S1 — Search Terms. (DOCX) [file pone.0084323.s002.docx]

**Appendix 1: Search Terms**

| **Setting** | **Intervention** | **Social Science Methods** |
| --- | --- | --- |
| Mental Disorder* OR Psychiatric Diagnos* OR Behavio$r Disorder* OR Reactive Disorder* OR Adjustment Disorder* OR Reactive Depression* OR Anxiety Disorder* OR Anxiety Neuros* OR Agoraphobia* OR Obsessive Compulsive Disorder* OR Panic Disorder* OR Panic Attack* OR Phobic Disorder* OR Phobic Neuros* OR Phobia* OR School Phobia* OR Social Phobia* OR Claustrophobia* OR Separation Anxiety OR Separation Anxiety Disorder* OR Mood Disorder* OR Affective Disorder* OR Depressive Disorder* OR Depressive Neuros* OR Endogenous Depression* OR Depressive Syndrome* OR Neurotic Depression* OR Melancholia* OR Unipolar Depression* OR Major Depressive Disorder* OR Dysthymic Disorder* OR Neurotic Disorder* OR Psychoneuroses | Computer Assisted Therap* OR Computer Assisted Psychotherap* OR Computer Assisted Counsel* OR Computer Assisted OR Computer Aided Psychotherap* OR Internet Therap* OR CCBT OR Internet Counsel* OR Internet Self Help OR Internet Self-Management OR Computer Based OR Inter-Active OR Interactive OR Automated OR Mobile Phone OR Online Therap* OR Telemedicine OR Telecommunication* OR Text Messaging* OR Email OR Electronic Mail OR Electronic Communication OR Virtual Reality OR VR OR Online OR Worldwide Web OR E-health OR Telemedicine* OR Teleconsultation* OR Telepsychiatry OR Telehealth OR Teletherap* OR Computer Mediated OR Remote Consultation OR Computerised Cognitive Behavio$r Therap* OR Computerized Cognitive Behavio$r Therap* OR Computerised CBT OR Computerized CBT OR Computer Supported OR Computer Assisted Intervention | Qualitative Research OR Interview* OR Ethnog* OR Case Stud* OR Grounded Theory OR Thematic Analysis OR Observational Method* OR Comparative Method* OR Field Notes OR Participant Observation OR Narrative* OR Field Stud* OR Audio recording OR Focus Group* OR Conversation* Analysis OR Descriptive Stud* OR Discourse Analysis OR Exploratory Stud* OR Hermeneutic OR Naturalistic OR Phenomenology* OR Participatory OR Open-ended OR In-depth OR Semi Structured OR Key Informant OR Tape Record* OR Cultural Anthropology Or Narration |
